# Study of the Influence of the Almond Shell Variety on the Mechanical Properties of Starch-Based Polymer Biocomposites

**DOI:** 10.3390/polym12092049

**Published:** 2020-09-08

**Authors:** Ana Ibáñez García, Asunción Martínez García, Santiago Ferrándiz Bou

**Affiliations:** 1 AIJU, Technological Institute for Children’s Products & Leisure Ibi, 03440 Alicante, Spain; sunymartinez@aiju.es; 2 Technological Institute of Materials (ITM), Universitat Politècnica de València (UPV), Plaza Ferrándiz y Carbonell 1, 03801 Alcoy, Spain; sferrand@mcm.upv.es

**Keywords:** almond shell, almond variety, biodegradable polymer, starch-based materials biocomposite, natural filler, biomass, injection moulding, mechanical properties

## Abstract

This article is focused on the development of a series of biodegradable and eco-friendly biocomposites based on starch polymer (Mater-Bi DI01A) filled with 30 wt% almond shell (AS) of different varieties (Desmayo Rojo, Largueta, Marcona, Mollar, and a commercial mixture of varieties) to study the influence of almond variety in the properties of injected biodegradable parts. The different AS varieties are analysed by means of Fourier transform infrared spectroscopy (FT-IR), thermogravimetric analysis (TGA), Scanning Electron Microscopy (SEM), and X-ray Diffraction (XRD). The biocomposites are prepared in a twin-screw extruder and characterized in terms of their mechanical (tensile, flexural, Charpy impact, and hardness tests) and thermal properties (differential scanning calorimetry (DSC) and TGA). Despite observing differences in the chemical composition of the individual varieties with respect to the commercial mixture, the results obtained from the mechanical characterisation of the biocomposites do not present significant differences between the diverse varieties used. From these results, it was concluded that the most recommended option is to work with the commercial mixture of almond shell varieties, as it is easier and cheaper to acquire.

## 1. Introduction

One of the greatest environmental challenges today is to find novel ways to utilise waste and residues derived from agricultural processes. The possibility to utilise biomass residues as fillers in polymer composites has attracted substantial interest, especially during the present decade. Various natural fillers such as jute, banana, alfa, argant shell, rice straws, or coffee grounds have been tested as reinforcement in polymer composites based on commodity plastics such as polyethylene (PE) or polypropylene (PP) [1]. This increased interest is due to their advantages over synthetic and mineral fillers: low cost, low density, non-toxicity, high specific properties, non-abrasive during processing, recycling possibility, and easy processability.

The components of natural fibre include cellulose, hemicelluloses, lignin, lipids, proteins, simple sugars, starches, water-soluble substances, hydrocarbons, ash, and small amounts of alkali, alkaline, and heavy metals and other compounds [2]. Depending on the content of the main components, cellulose, hemicellulose and lignin, the fibres will present different properties. For example, a high concentration of hemicellulose provides high thermal stability to natural fibres, but mechanical properties are reduced and, consequently, so are the mechanical properties of the biocomposites. A high content of cellulose improves the mechanical properties as opposed to biological degradation and resistance to moisture decrease. Regarding lignin, this component has less influence on mechanical properties, as well as thermal and biological degradation. However, a high concentration of lignin favors the photodegradation phenomena [3].

The chemical composition of natural fibres varies according to the types, variety, age, climate, geographic region, soil conditions, and even between the different parts of a plant [1]. The information on the chemical composition of natural fillers and fibres is important because it determines their properties [3]. Table 1 shows the content of the main components, cellulose, hemicellulose and lignin, of some types of natural fibres.

**Figure 1:**
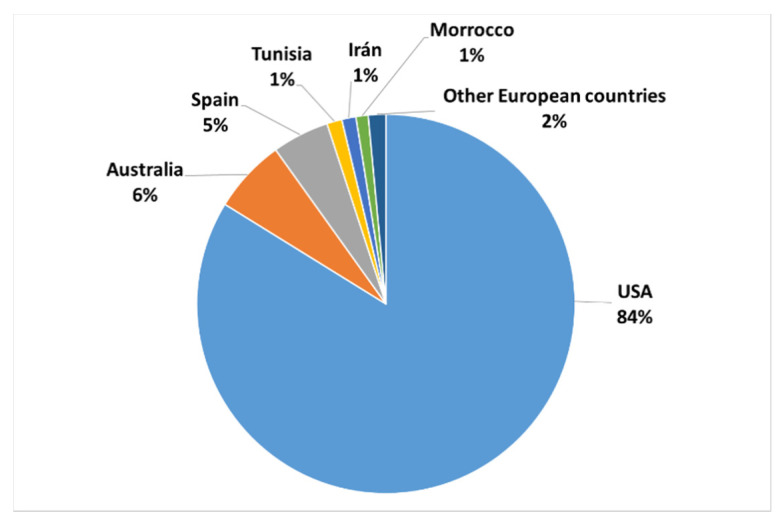
Production of almonds by country.

Almond shell is a type of biomass that is obtained from the cultivation of the almond. Almond cultivation is spread in many regions of the world, being the main producer the United States, followed at a great distance by Australia, Spain, Tunisia, Iran, and Morocco (Figure 1). These countries are joined by Greece, Turkey, and Italy, which complete the list of other European producers, according to data presented at the 37th International Congress on Nuts [4].

Almond shells represent around 65–80% of the total weight of the fruit [5], so with only the Spanish production of almonds, in the 2018/2019 campaign, there were between 205,000 and 250,000 tons of shells. There are a huge range of varieties of almonds and they can be distinguished according to their taste, use, flowering time, hardness shell, etc. Traditionally, the use of almond shells has been as food for animal husbandry or for obtaining energy from biomass cogeneration [6,7]. However, almond shells are an increasingly abundant waste product, and their high availability has sparked great interest in using this by-product as an absorbent for heavy metals and dyes, compost, production of active carbons, and production of xylo-oligosaccharides [8,9].

Nowadays, research efforts are centred in developing composites by combining natural fillers with biodegradable resins [10]. Thus, some researches on poly(lactic acid) (PLA) [11,12,13,14,15,16], polyhydroxybutyrate (PHB) [17,18,19,20], starch-based polymer (TPS) [21,22,23,24,25], polybutylene succinate (PBS) [26,27,28,29,30] or polycaprolactone (PCL) [31] with different natural fillers/fibres have been reported. In general, it was observed that tensile and flexural modulus increased, but the impact strength and elongation at break decreased. As is known in the field of biocomposites, the greatest challenge in working with natural fibres is their large variation in properties and characteristics. Their properties and processing of composites, which are reinforced by natural fibres, are affected by many factors such as the chemical composition, morphology, size, dispersion/distribution, interfacial adhesion, and particle content [32]. The majority of researchers have focused their interest to study the effect of size and fibre content on mechanical properties [33,34,35]. They found that an increase in fibre size produced higher tensile strength and modulus of elasticity, while impact strength and elongation at break were decreased. Furthermore, the higher the natural fibre content, the higher the strength, stiffness, and lower impact strength. Other studies analyse the effect of using different types of natural filler/fibre on the matrix polymer [25,36]. However, no studies have been found that analyse the influence of the different varieties of the same type of natural fibre on the properties of the biocomposite. As it was mentioned before, the chemical composition of natural fibres can vary according to variety, and this determines their properties and the biocomposite properties. Since the availability of this type of natural fillers is usually a mixture of the different varieties, it has been considered relevant to check and study if the use of one or another variety could influence the final properties of the biocomposites to which it is added, so that this is not a handicap when it comes to implementing this biomaterial at an industrial scale. No previous studies have been found in this aspect, neither in the case of almond shells nor other varieties of lignocellulosic fibres or fillers from plants; thus, this work contributes to increasing the present state of knowledge.

Biodegradable plastics offer important contributions by reducing the dependence on fossil fuels. Among the biodegradable materials, the starch-based polymers are considered of high interest to develop sustainable materials for consumer applications, due mainly to their low cost, complete biodegradability [37], and renewability [38]. One of the most popular commercial TPS is Mater-Bi^®^, which is a family of modified biodegradable and compostable thermoplastic starches produced by Novamont [39]. Different grades of Mater-Bi^®^ exist, and the main difference between them is their composition. Depending on material composition, it presents different properties. It is possible to find blends of starch with cellulose acetate (AC), polycaprolactone (PCL), or polybutylene adipate-co-terephthalate (PBAT), among others. There are different developments of biocomposites based on thermoplastic starch with a wide variety of natural fibres/fillers such as hemp [40,41], bagasse [42], coir [21], bamboo [24], sisal [43,44], wheat straw fibres [45], wood [22], and alpha fibres [46]. It was shown that natural fillers or fibres can be successfully incorporated into the Mater-Bi^®^ matrix, independent of the grade. In general terms, the addition of natural filler/fibres increases the mechanical properties, but this depends on different factors as particle size, particle content, and interfacial adhesion between polymer–filler. At the same time, natural fillers/fibres can act as thermal stabilisers, for example in the case of hemp and kenaf fibre [25].

At present, the main areas of application of biomaterials and biocomposites are packaging, catering, agriculture and automotive. In addition, the bioplastics and biocomposites industry is becoming more present in the children’s sector, from toys to childcare products. The combination of natural fibres or wood flour mixed with biodegradable or bio-based plastics is a potential and attractive alternative for these traditional industries where new millennial consumers are more respectful with the environment, and parents want their children to acquire an ecological vision of the world [47].

The main objective of the present study is to develop and characterise biocomposites based on a commercial starch-based thermoplastic matrix, Mater-Bi^®^, which is filled with almond shells of different varieties. This paper also studies the influence on the properties of the polymeric and checks that the mixture of varieties can be used even if the proportion of varieties changes a little so that quality problems in the biocomposites do not appear at an industrial scale. This study provides the ground to critically select the most promising almond shell variety to be used as a filler.

## 2. Materials and Methods

### 2.1. Materials

A commercially available starch-based polymer, Mater-Bi DI01A of Novamont, was used in this study. This bio-based and biodegradable polymer has a melt flow index (MFI) of 35 g/10 min (190 °C/2.16 kg) and a density of 1200 kg/m^3^ (data provided by Novamont). There is little scientific information available about it, but it is known that it is mostly based on a TPS and PBAT blend. This reference was selected for their properties similar to polypropylene (PP). Mater-Bi DI01A possesses high renewable content up to 80%. Table 2 shows some properties of the as-received material.

**Figure 2:**
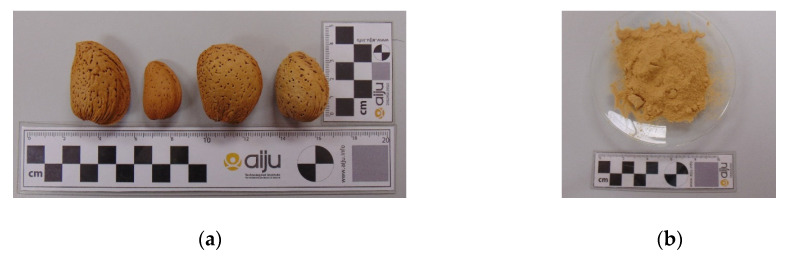
(**a**) Almond shell varieties studied: Desmayo Rojo, Largueta, Marcona, Mollar (from left to right), (**b**) Commercial mixture powder.

A common classification of almond varieties is by the type of shell, so two types of almonds can be distinguished: those with a soft shell and those with a hard shell. It was considered of interest to study if this characteristic could influence the final properties of the biocomposites to be developed; therefore, four types of almond varieties were selected for the study: Desmayo Rojo, Largueta, Marcona (hard shell), and Mollar (soft shell) [48], all of them from Spain (Figure 2). Since both the separation of the almond varieties in the field and of the shells in the cracking/shelling plants is complicated, the most usual format of acquiring this waste in the market is as a mixture of shell varieties. Then, biocomposites were also developed by using a mixture of different varieties in order to compare the results with the separated ones. The mixture was provided by Hermen Europe, S.L in the form of powder with a particle size between 0.05 and 0.125 mm.

### 2.2. Experimental Procedure

#### 2.2.1. Milling of Almond Shell

Prior to processing, almond shell powder (ASP) was obtained by milling in two steps, using a Milling Shini model SG-1621 (size particle less than 5 mm) and Milling ZM 200 (size particle less than 1 mm). The resultant powder was sieved using a set of sieves to obtain different size particles (0.250–0.125, 0.125–0.08, 0.08–0.05, and less than 0.05 mm).

The moisture content of almond shell is around 10–13 wt%. It can drastically affect the processing, producing hydrolytic reactions [46] and weakening some of the mechanical characteristics of the biocomposites to be developed, such as tensile/flexural properties [49,50,51] and impact strength. Therefore, the almond shell powder was dried to minimise its moisture content. This was carried out in an air-circulating oven for 24 h at 105 °C before processing. The moisture content of the ASP after the drying process was less than 1 wt%.

#### 2.2.2. Infrared Spectroscopy (FTIR)

The infrared spectra of each almond variety and the mixture were recorded on a NICOLET NEXUS 6700 Spectrophotometer. The KBr disk method was employed, analysing the powder samples of the different varieties of almond shells particles of 0.125–0.250 mm and mixture. The spectra were obtained at an angle of incidence of 45°, and the transmittance range of the scan was 400 to 4000 cm^−1^, recording 32 scans, using the attenuated total reflection (ATR) accessory on the KBr disk.

#### 2.2.3. Thermogravimetric Analysis (TGA)

The biochemical composition of different almond shell varieties, the mixture, and the thermal stability (degradation/decomposition) of biocomposites developed were determined by thermogravimetric analysis (TGA) using a TA Instrument Q500 thermogravimetric analyser (TGA). Samples with an average weight comprised between 8 and 10 mg were placed in standard alumina crucibles of 70 µL.

For determining the content of fixed carbon, volatile matter, humidity, and ash content, all almond shells are subjected to a constant heating rate of 20 °C/min from 30 to 120 °C under an N_2_ controlled flow of 100 mL/min. The samples were kept for one hour at 120 °C to remove the moisture content. Then, they were heated to 800 °C to determine the volatile matter content. Immediately, the gas flow was changed to air to gasify all carbonaceous material in order to quantify the fixed carbon and to obtain the ash content.

The main thermal degradation parameters of biocomposites, degradation initial temperature (T_onset_), and temperature for maximum mass loss rate (T_max_) were also studied by TGA. In this case, biocomposites were subjected to the following temperature program: from 30 to 600 °C under an N_2_ atmosphere at a rate of 10 °C/min, and from 600 to 1000 °C under an O_2_ atmosphere at a rate of 10 °C/min with a purge gas flow of 10 mL/min.

#### 2.2.4. Differential Scanning Calorimetry

Thermal transitions of developed biocomposites were studied by differential scanning calorimetry (DSC) in a DSC Q200 calorimeter for TA Instruments with a heating program from 0 to 220 °C in nitrogen atmosphere (50 mL/min) at a heating rate of 10 °C/min.

#### 2.2.5. X-ray Diffraction

The different varieties of almond shell particles of 0.125–0.250 mm and the mixture were measured by X-ray diffraction (D/max-2200VPC, Japan Science Co., Ltd., Takatsuki City, Osaka, Japan) in the range of 2θ = 5°–40° at scanning speed of 5 rad/cm. The X-ray source was a Cu target (Cu Kα = 1.54056), and a nickel filter was used to excite alpha radiation. The crystallisation index of the sample was calculated according to the following formula [5]:

(1) $$CrI\;\left(\%\right)=\frac{I_{002}-I_{am}}{I_{002}}\times 100$$

where *I*_002_ is the intensity at 2θ = 22°, and *I*_am_ is the intensity of background scatter at 2θ = 16°.

#### 2.2.6. Scanning Electron Microscopy (SEM)

The morphological structure and size of almond shell particles and impact fracture surface obtained after a Charpy impact test were analysed using a Jeol JSM-840 SEM system. Almond shell powder samples were gold-coated before analysis, and the energy of the electron beam was 20 kV.

#### 2.2.7. Preparation of Composites

Composites of starch-based polymer and ASP were developed using a Rondol co-rotating twin-screw extruder (20:1 L/D) with 10 mm diameter. The polymer was fed through the main hopper and the ASP was fed through a secondary hopper.

In a previous study, in the process of publication, the effect of the particle size of almond shell powder (ASP) filler on starch-based biodegradable polymer was studied. Different size ranges of particles were studied: <0.05 mm, 0.05–0.08 mm, 0.08–0.125 mm, and 0.125–0.250 mm in biocomposites with 30 wt% ASP. Additionally, biocomposites with 5 wt%, 10 wt%, and 20 wt% of filler were prepared with ASP of 0.08–0.125 mm particle size. The higher the natural fibre content, the higher strength and stiffness and the lower impact strength. In this work, almond shell content was added to the starch-based biodegradable polymer in 30 wt%. The composition of the almond shell filler was made up of different particle size powders of the same variety (0.250–0.125, 0.125–0.08, 0.08–0.05, and less than 0.05 mm) in equal proportion (25% each).

The temperature profile was set as follows: 130–185–185–185–185 °C (from feeding zone to die). A rotating speed was 74 rpm. The extruded materials were finally pelletised using an air-knife.

#### 2.2.8. Injection Moulding

Testing samples were moulded using an injection moulding machine MTT 12/90 HSE. The injection conditions used to prepare test samples are shown in Table 3. Finally, specimens were conditioned at a temperature of 23 °C and relative humidity of 50% for at least 16 h before testing.

#### 2.2.9. Tensile Strength and Modulus

Tensile testing of the injection-moulded composite specimens was performed with an Instron 6025 universal testing machine with 5 kN power sensors. The tests were performed according to standard ISO 527, starting with a crosshead speed of 1 mm/min, accelerating to 5 mm/min when the strain exceeds the 0.25 mm limit. The extensometer used was MTS 634.11F-54. Recorded values include ultimate tensile strength (UTS), Young’s modulus, and strain at break. A total of 5 specimens from each material were tested using standardised samples 1BA (dogbone).

#### 2.2.10. Impact Strength

Impact testing was performed with a Resil 5.5 impact testing device (CEAST RESILIMPACTOR) with a 1 Joule hammer. Test samples were cut and tested according to standard ISO 179 (Charpy un-notched). A total of 5 specimens from each material were tested.

#### 2.2.11. Flexural

Flexural testing was performed with an Instron 6025 universal testing machine with 5 kN power sensors. The flexural strength and the flexural modulus of elasticity were determined as a three-point bend. The test speed was 2 mm/min. The flexural strength was calculated according to standard ISO 75. The test was run with five specimens using standardised samples of 80 mm^3^ × 10 mm^3^ × 4 mm^3^.

#### 2.2.12. Shore D Hardness

The hardness of composites was measured with Shore D hardness tester, BAREISS B5-61, in accordance with ISO 868. The hardness was measured at different points on specimen.

## 3. Results and Discussion

### 3.1. Characterisation of Almond Shell Varieties

**Figure 3:**
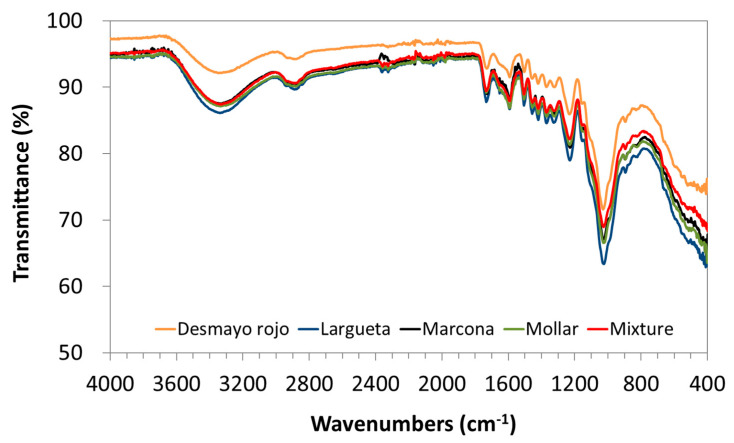
Fourier transform infrared spectroscopy (FT-IR) spectrums of almond shells varieties: Desmayo Rojo (**orange**), Largueta (**blue**), Marcona (**black**), Largueta (**red**), Mollar (**green**), mixture (**red**).

The chemical characterisation of the different almond shell varieties and the mixture was performed by FT-IR analysis, and their corresponding spectra are shown in Figure 3. Table 4 summarises the main peaks of almond shells. The spectrum of the almond shell shows the basic structure of all lignocellulosic fibres, i.e., strong broad OH stretching vibrations (3300–4000 cm^−1^) due to intermolecular hydrogen banding of polymeric compounds (macromolecular associations) such as alcohols, phenols, and carboxylic acids, and, as in pectin, cellulose groups on the adsorbent surface. The peaks at 2916 and 2852 cm^−1^ are attributed to the symmetric and asymmetric CH stretching vibration of aliphatic acids. The peaks around 1395 cm^−1^ are due to the symmetric bending of CH_3_. The peak observed at 1630 cm^−1^ is the stretching vibration of bond due to non-ionic carboxyl acids or their esters. The broad peak at 1072 cm^−1^ may be due to the stretching vibration of C–OH of alcoholic groups and carboxylic acids [3]. No important differences are appreciated between the almond shells from different almond varieties.

**Figure 4:**
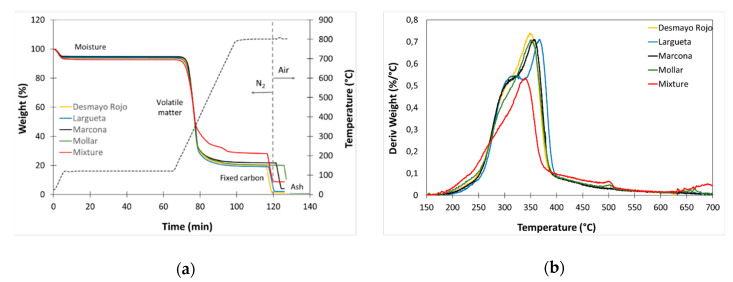
(**a**) Thermogravimetric curves of almond shells. (**b**) First derivative (DTG) curves of almond shell. DTG: differential thermogravimetric curves.

Thermogravimetric analysis (TGA) was used to determine the content of fixed carbon, volatile matter, humidity, and ash of lignocellulosic or carbonaceous materials [52]. Figure 4a represents the thermogravimetric curves for the different types of almond shells and the mixture of varieties, and Table 5 collects the results corresponding to the content of moisture, volatile matter, fixed carbon, and ash obtained. All samples show typical values for this type of lignocellulosic biomass, with a low amount of fixed carbon and a large amount of volatile matter. In turn, it is evident that the Molar, Largueta, and Desamayo Rojo varieties contain a lower ash content compared to Marcona and the mixture of varieties. Figure 4b shows the differential thermogravimetric curves (DTG) obtained in the decomposition temperature range of volatile matter. As observed, the five samples show differences in the decomposition profiles, evidencing the differences in their composition.

**Figure 5:**
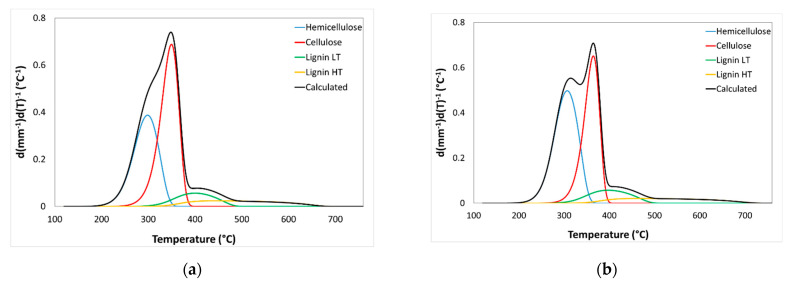

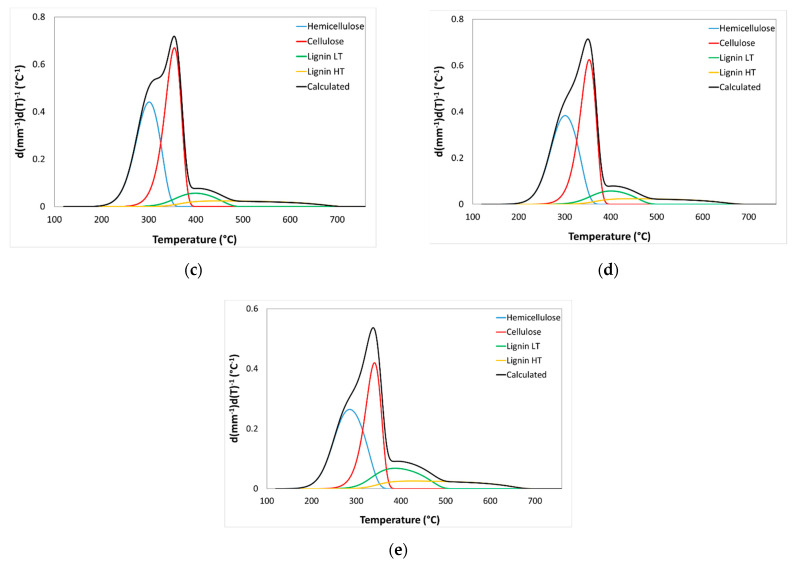
Comparison between experimental DTG profiles and simulated differential curves (black line) including the deconvolutions obtained by means of an independent parallel reaction model for all almond shells varieties analysed: (**a**) Desmayo Rojo, (**b**) Largueta, (**c**) Marcona, (**d**) Mollar, (**e**) mixture.

In addition, this technique is used to study the decomposition of the different components of lignocellulosic biomass—hemicelluloses, cellulose, and lignin [2,40]—and determine the kinetic parameters, such as activation energy (*E*_a_), pre-exponential factor (*K*_0_), and pyrolysis heterogeneity factor (α). Table 6 shows the kinetic parameters obtained from the deconvolutions carried out on the DTG curves (Figure 5). These kinetic parameters are in agreement with those published [53,54,55,56,57]. From the area obtained in the integration of each curve associated with the decomposition of the biopolymers, the biocomposition of each of the samples is determined considering their carbonisation performance (Table 6). In the case of lignin decomposition, due to the high heterogeneity of the reaction, the calculation is carried out using two independent pyrolyses: the first one for lignin decomposes at low temperatures (LT) and the second one for lignin decomposes at high temperatures (HT), the second one being the most heterogeneous.

In general terms, all the almond shells analysed present typical values of lignocellulosic biomass or common shells from different agricultural crops, i.e., groundnut, hazelnut, pistachio, and walnut [5,58], showing cellulose contents between 32% and 40% for all the samples; except for the mixture sample, that presents values below 30%. In turn, the hemicellulose content is similar in all varieties, obtaining values between 36% and 44%. However, it is observed that such hemicellulose has a more heterogeneous character in samples 2 and 5. In the case of lignin, the Desmayo Rojo, Largueta, Marcona, and Mollar varieties have equal contents and similar characteristics, contrary to the mixture sample, which has a higher quantity of this component, being in turn more heterogeneous. This could because the mixed sample has traces of other parts of the almond tree—for example, leafs, branches, etc. or other substances from the milling process.

**Figure 6:**
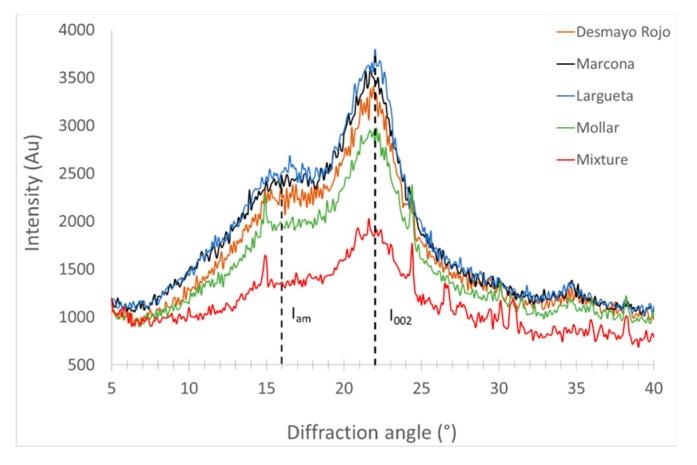
X-ray diffraction spectra.

A high content of cellulose in natural fibres improves the mechanical properties due to higher crystallisation because cellulose is responsible for crystallisation. For composites, the application of a crystallinity index result could be considered as the factor for choosing that fibre during composite manufacturing. The XRD analysis determines the crystallinity index of natural fibres. Figure 6 shows the X-ray diffraction curves of different varieties of almond shell. As it can be seen in Table 7, the different varieties studied present some differences. The Largueta variety presents a crystallinity index of 33.8%, which is the highest of the varieties studied, while the mixture is 25.1%, the lowest out of all of them. The results obtained from almond shell varieties separately are in accordance with the obtained in previous studies [59]. The crystallisation values follow the same trend as in TGA analysis for the different varieties—that is, the commercial mixture with a lower cellulose content exhibits a lower crystalline index than Desmayo Rojo, Largueta, Marcona, and Mollar with higher cellulose content.

**Figure 7:**
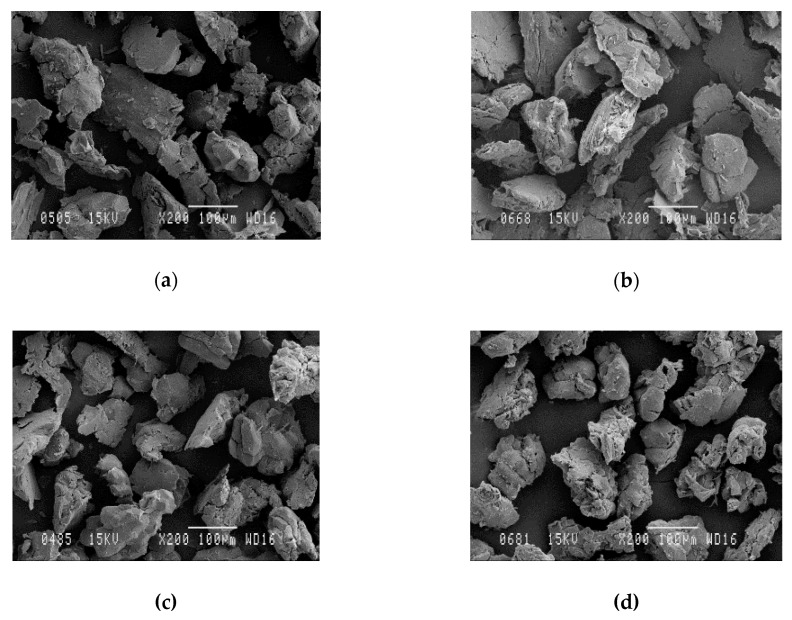
SEM micrographs (×200 magnification) of almond shell: (**a**) Desmayo Rojo variety, (**b**) Largueta, (**c**) Marcona, and (**d**) mixture of all.

**Figure 8:**
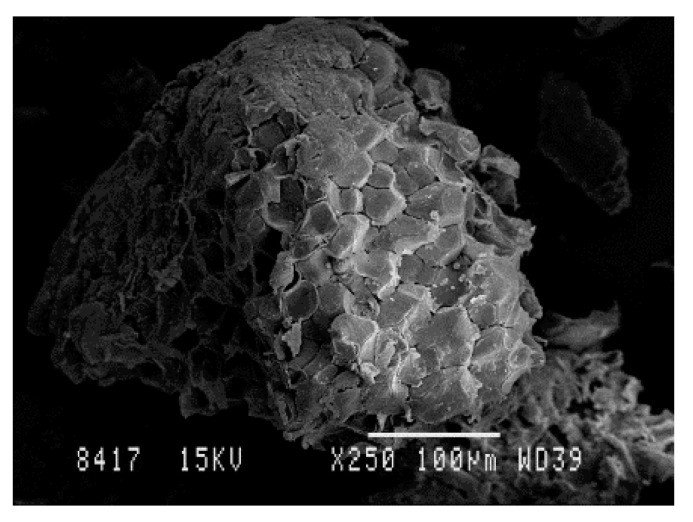
Almond shell (×250 magnification) Marcona variety, 0.250–0.500 mm particle size.

The morphology of almond shells was studied by SEM. Figure 7 shows the images of particles between 0.08 and 0.125 µm size, of the different varieties. As can be appreciated, there are no significant differences in the morphological structure. SEM micrographs also show the agglomeration of many fine microparticles, which led to a rough surface and the presence of pores in the structure (Figure 8) A similar morphology for ASP particles has been reported in previous studies of polymer composites filled with almond shells [12].

### 3.2. Development and Characterisation of the Biocomposites

**Figure 9:**
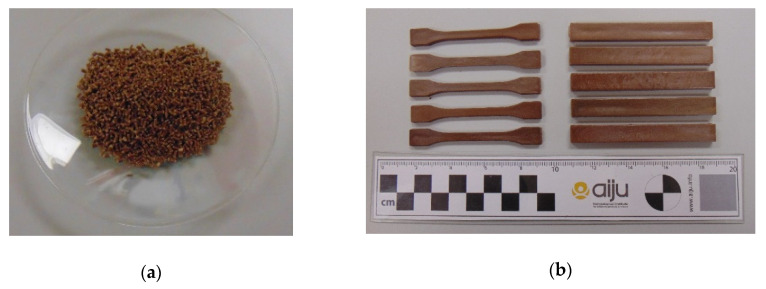
(**a**) Pellets of Mater-Bi DI01A with 30 wt% almond shell; (**b**) Injected samples of Mater-Bi DI01A with 30 wt% almond shell.

The extrusion compounding process was correct. The filament extruded was pelletised, obtaining the pellet (Figure 9). The materials developed were injected according to the conditions indicated in Table 2 in order to obtain testing samples. The biocomposites obtained with 30 wt% almond shell present a brown colour. The almond shell variety added does not substantially influence the aesthetic result.

**Figure 10:**
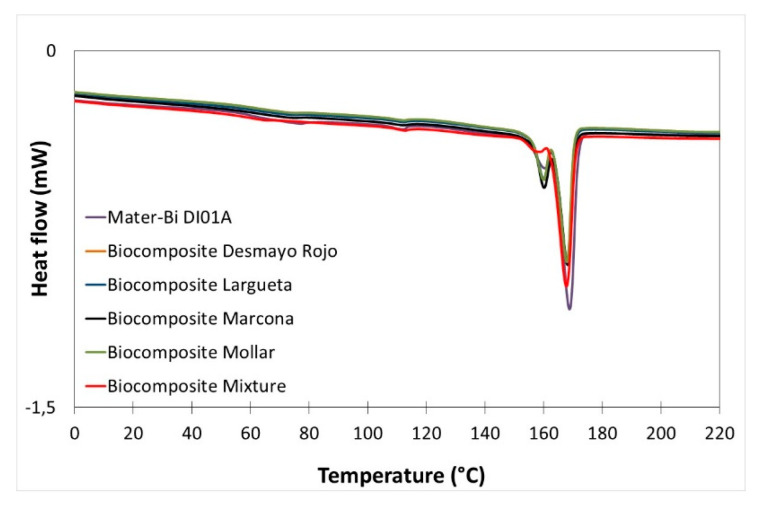
Comparative plot of the DSC curves starch-based polymer (Mater-Bi DI01A) and starch-based polymer/ASP biocomposites with different almond shell varieties.

Differential scanning calorimetry was used to obtain the main thermal transitions of the as-received polymer and the developed biocomposites (Figure 10). Table 8 shows the main thermal parameters obtained through DSC characterisation. As it can be observed, the addition of 30 wt% of almond shell produces a slight reduction in the melting point temperature (Tm) of about 1 °C, from 168.84 °C for as-received starch-based polymer down to 167.70 °C for the biocomposite with a mixture of varieties. A small peak can be observed around 159–161 °C. This may be attributed to the beginning of fusion of crystalline structure. With regard to the normalised melting enthalpy (ΔH_m_), it decreases from 28.01 to 21–22 J/g. These results may indicate that the addition of almond shell to the starch-based polymer slightly decreases the crystallisation of the molecular chains. These calorimetric results are consistent with previously reported effects in similar works where almond shell is added to other biodegradable matrixes such as PLA [11] or PBS [26].

**Figure 11:**
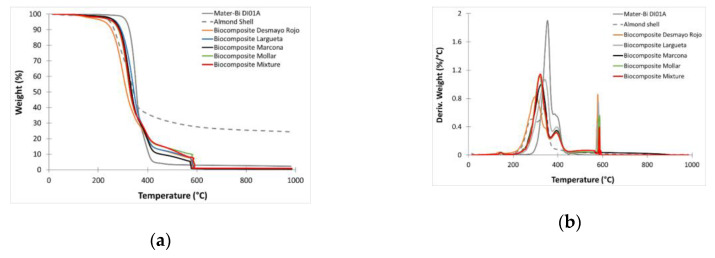
(**a**) Thermogravimetric analysis (TGA) thermograms corresponding to starch-based polymer/ASP biocomposites with different almond shell varieties; (**b**) first derivative (DTG) curves.

Figure 11 shows the TGA and DTG curves of the almond shell powder, as well as that of the as-received polymer, and the starch-based polymer/almond shell composites with different varieties. The as-received starch-based polymer degrades in a single step, and its T_onset_ is close to 325.98 °C, thus indicating moderate thermal stability. The addition of almond shell particles reduces the thermal stability of the biocomposites because almond shells start degradation earlier than polymer matrix. T_onset_ and T_max_ are moved towards lower temperature with the addition of almond shell. As shown in Table 9, T_onset_ changes progressively from 325.98 °C for as-received starch-based polymer, decreasing to values of 281.00 °C. There are some differences between the varieties studied. This is explained because the thermal stability of the natural fibre depends on the chemical composition (hemicellulose, cellulose, and lignin) and specifically, with hemicellulose content. The higher content of hemicellulose, the higher the thermal stability. The biocomposite based on the Largueta variety presents the highest value of T_onset_ and T_max_; this variety is the one that presents higher hemicellulose content.

**Figure 12:**
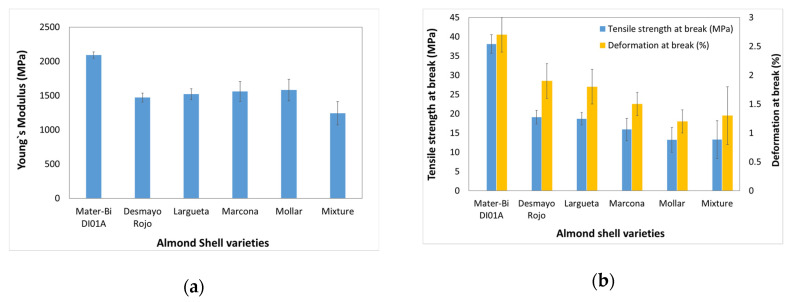
Tensile properties of as-received Mater-Bi DI01A and biocomposites with different almond shell varieties (**a**) Young’s modulus; (**b**) Tensile strength, and deformation at break.

Figure 12 shows the Young’s modulus, tensile strength at break, and deformation at break of the developed samples. In previous studies, the addition of the natural fibres—i.e., cotton, hemp, or kenaf in starch-based polymer—increases the tensile modulus [23]. The tensile strength of composites is related to interfacial strength, and thus, if there is weak strength in the interface, the tensile strength of the composites will be low. As it can be seen, the starch-based polymer presents a Young’s modulus of 2100 MPa. The biocomposite specimens developed showed a decrease in the Young’s modulus, reaching the lowest value 1240 MPa with the biocomposite based on a mixture of varieties. Besides, there is a noticeable decrease in the flexural strength and the flexural strain. The as-received starch-based polymer specimens present a flexural strength of 59.3 MPa and a flexural strain of 4.5% down to 13.2 MPa and 1.2%, respectively, in the case of the biocomposites based on Mollar and Mixture varieties. This behaviour gives clear evidence that the polymer–particle cohesion is poor and, also, the fibre can act as stress concentrators. Besides, the lower tensile values are obtained with the biocomposites with the variety that has a lower crystallinity index.

**Figure 13:**
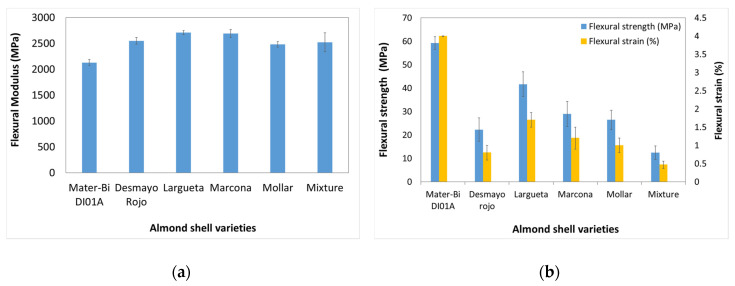
Flexural properties of as-received Mater-Bi DI01A material and biocomposites with different almond shell varieties: (**a**) Flexural modulus; (**b**) Flexural strength and flexural strain.

The effect of the variety of almond shells was evaluated by flexural tests (Figure 13). The incorporation of almond shells increases the flexural modulus. The as-received starch-based polymer presented a flexural modulus of 2130 MPa. The biocomposite specimens showed an increase in the flexural modulus, reaching the highest value, 2710 MPa, in the biocomposite based on the Largueta variety with the highest cellulose content. A similar value presents the composite with the Marcona variety. However, it can be observed a noticeable decrease in the flexural strength and the flexural strain. The as-received starch-based polymer specimens present a flexural strength of 59.3 MPa and a flexural strain of 4.5% down to 12.5 MPa and 0.5%, respectively, in the case of the biocomposites based on a mixture variety. This effect could be due to the poor biopolymer–filler interfacial adhesion. The highest values in flexural strength and strain were presented by the Largueta variety and the lowest were presented by the mixture variety.

**Figure 14:**
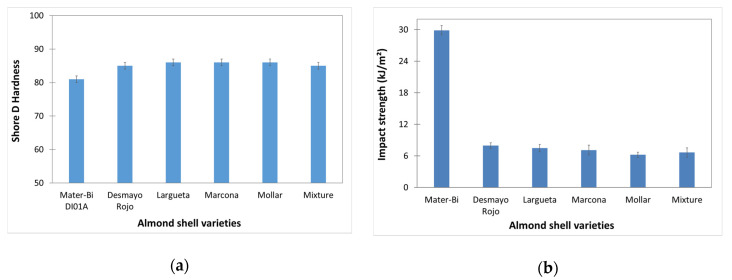
(**a**) Shore D hardness; (**b**) Impact strength corresponding to starch-based polymer/ASP biocomposites with different almond shell varieties.

The Shore D hardness values are shown in Figure 14a. The incorporation of almond shell slightly increases the Shore D hardness of the material. Specifically, it increased from 81 to 86, which can be related to the reinforcing effect of a hard filler on the biopolymer matrix.

Impact properties are the most affected ones by the addition of ASP, as shown in Figure 14b. The addition of ASP drastically reduces the impact strength. While the as-received starch-based polymer specimens showed an impact strength of 29.9 kJ/m^2^, the biocomposite specimen presented a value between 6.2 and 7.9 kJ/m^2^. This fact can be related to the high content of ASP (30 wt%), which potentially produced high tensile stresses and a very low deformation degree along the part, leading to a low-impact energy absorption [11]. Similar results were obtained in previous studies, in which alpha, bagasse, or hemp fibre was added to a starch-based polymer matrix between 15 wt% and 20 wt%. However, comparing the almond shell varieties studied, there are not significant differences. The highest impact strength value was presented by the biocomposite based on Desmayo Rojo and Largueta, which had a higher cellulose content.

**Figure 15:**
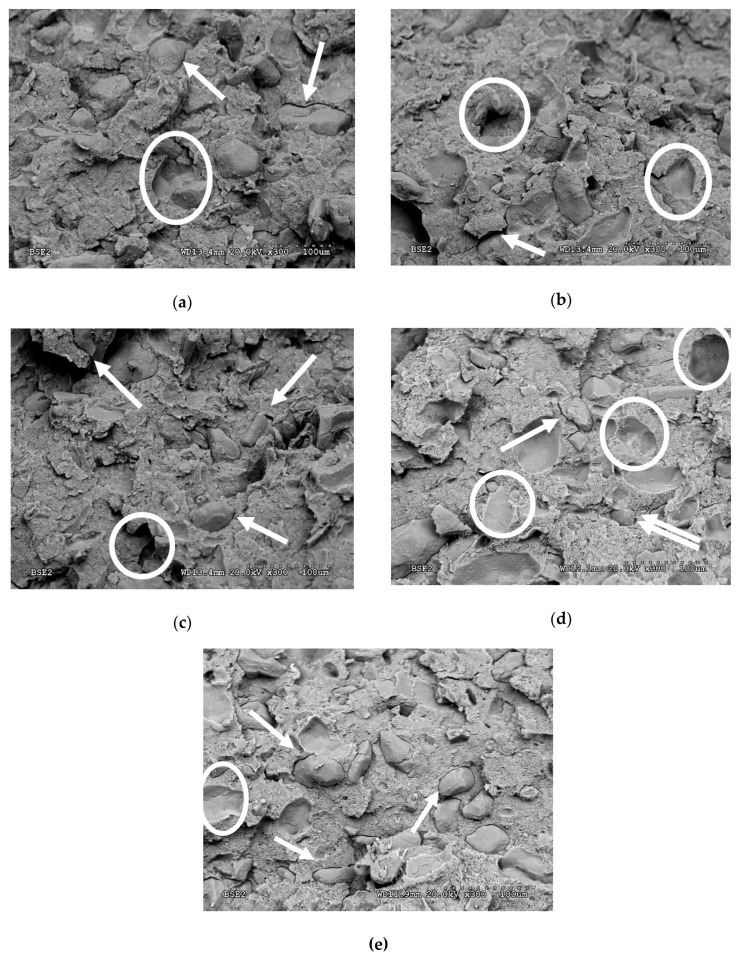
SEM micrographs of the impact fracture surfaces of the composites with different almond shell varieties: (**a**) Mater-Bi DI01A/Desmayo Rojo; (**b**) Mater-Bi DI01A/Largueta; (**c**) Mater-Bi DI01A/Marcona; (**d**) Mater-Bi DI01A/Mollar; and (**e**) Mater-Bi DI01A/Mixture of varieties.

The fractured surface of the biocomposites after impact tests were studied by SEM. The obtained micrographs (Figure 15) show that the almond shell is homogeneously distributed in the thermoplastic polymer matrix. A gap between almond shell particles and the surrounding starch-based polymer matrix can be observed (see arrows). In addition, the impact fracture surface seemed to have several voids (see circles) that would correspond to the detached particles after impact, indicating a poor interfacial adhesion between ASP and the polymeric matrix. This supports the impact strength results obtained previously, showing that the biocomposites presented brittle behaviour compared to the as-received material. Several studies of biocomposites based on biodegradable matrices, such as PLA, PBS, starch polymer, and natural fibres show that the application of chemical treatments to nature fibre [21] or adding additives (coupling agents [26], epoxidised oils [60]) can improve the processability, the matrix/fibre compatibility, and the ductile mechanical properties of the biocomposites.

## 4. Conclusions

It has been stated that the incorporation of 30% ASP into biodegradable starch-based polymers produces aesthetic, mechanical, and thermal changes. Formulations were successfully processed by melt extrusion followed by the injection-moulding process. The processing equipment used on a laboratory scale is analogous to that used on an industrial scale. In fact, for later works, industrial equipment was used without any problem, collaborating with real compounding companies and injection moulders working in the toy, household, and packaging industries (Project MASTALMOND LIFE11 ENV/ES/513). It was not necessary to adapt the line with new devices or additional equipment.

The analysis of the chemical composition of the five samples of almond shells studied has shown differences between them. As it is collected in related studies in the field of biocomposites with natural fillers or fibres, the properties of these present are closely related to their chemical composition, and this can vary according to the type, variety, age, climate, geography, etc. However, this study showed that there are no substantial differences in the mechanical and thermal properties of biocomposites developed using different varieties of almond shells. Therefore, the most recommendable option is to work with the commercial mixtures of almond shell varieties, since the resulting biocomposites present mechanical properties similar to those of other varieties of almond shells separately. Moreover, nowadays, this is available in the supply chain as a mixture of those already crushed, which makes it easier and cheaper to acquire. This is because the work of separating by variety and crushing would make the resulting product, the almond shell powder, more expensive. Nowadays, commercial powder of almond shell (mixed varieties) is available in the market; for example, some suppliers of mixture of almond shell varieties powder are Hermen S.L, Frupinsa, Alejandro Tapia, S.L, and Pellets del Sur.

The experimental results revealed that the incorporation of the filler slightly increases the rigidity of the material as demonstrated by the slight increase of the flexural modulus; however, the impact resistance and tensile and flexural strength at break decrease drastically. The almond shell varieties studied do not have a significant effect on the hardness and impact strength.

From the experience gained in processing and the results obtained, it was concluded that the new bio-based and biodegradable compounds can be used in different consumer applications to replace other fossil-based materials. However, since the ability of biocompound samples to absorb energy was significantly compromised, this should be carefully considered for high-tech applications and/or further research to improve the formulations. Currently, the effects of different epoxidised vegetable oils (EVOs), such as epoxidised linseed oil (ELO), epoxidised corn oil (ECO), and epoxidised soybean oil (ESBO), are being studied on starch polymer/ASP biocomposites. The first results obtained show an improvement in the processability, the matrix/fibre compatibility, and the ductile mechanical properties of the biocomposites.

## Figures and Tables

**Table 1:** Chemical composition of different natural fibre [1].

Natural Fibre	Cellulose (%)	Hemicellulose (%)	Lignin (%)
Bagasse	55.2	16.8	25.3
Bamboo	26–43	30	21–31
Flax	71	18.6–20.6	2.2
Kenaf	72	20.3	9
Jute	61–71	14–20	12–13
Hemp	68	15	10
Ramie	68.6–76.2	13–16	0.6–0.7
Abaca	56–63	20–25	7–9
Sisal	65	12	9.9
Coir	81	--	12.7
Pinapple	81	--	12.7
Wheat straw	38–45	15–31	12–20
Rice husk	35–45	19–25	20
Rice straw	41–57	33	8–10

**Table 2:** Properties of MATER-Bi DI01A extract to datasheet supplied by Novamont.

Characteristics	TEST	Values
Min. processing temperature (°C)	Novamont test	170
Max. processing temperature (°C)	Novamont test	260
Melting temperature (°C)	ASTM-D3418	160
Melt viscosity (Pa·s) (T = 190 °C, γ = 1000 s^−1^)	ASTM-D3835	140
Tensile strength at break (MPa)	ASTM-D638	20
Max. tensile strength (MPa)	ASTM-D638	48
Elongation at break (%)	ASTM-D638	22
Elongation at max strength	ASTM-D638	2.5
Young modulus (MPa)	ASTM-D638	2700

**Table 3:** Injection condition of starch-based polymer and ASP biocomposites with different almond shell varieties.

Parameters	Injection Moulding Conditions
Injection temperature (°C)	40–180–190–200–200
Mould temperature (°C)	30
Injection speed (mm/s)	70
Injection pressure (max)(bar)	165
Back pressure (bar)	83
Cooling time (s)	35
Injection temperature (°C)	40–180–190–200–200
Mould temperature (°C)	30
Injection speed (mm/s)	70

**Table 4:** Absorption band assignment in the infrared spectrum of almond shell [5].

Wavenumber (cm^−1^)	Functional Group	Vibration Type	Cause
3300–3500	─OH	stretching vibration	cellulose, hemicellulose
2900–2935	─CH	stretching vibration	-
1640–1735	C=O	stretching vibration	lignin, hemicellulose
1580–1605	benzene ring	stretching vibration	lignin
1455–1465	─CH_3_O	stretching vibration	lignin
1320–1430	─CH	bending vibration	-
1221–1230	C─C C─O	stretching vibration	lignin
1025–1035	C─O	stretching vibration	cellulose, hemicellulose and lignin
885–895	R_2_C=CH_2_	bending vibration	-
810–833	benzene ring	disubstituted benzene	-

**Table 5:** Content of moisture, volatile matter, fixed carbon, and ash of different varieties of almond shells.

Almond Shell Variety	Moisture (%)	Volatile Matter (%)	Fixed Carbon (%)	Ash (%)
Desmayo Rojo	6.2	73.9	18.9	1.0
Largueta	5.8	75.1	17.1	2.0
Marcona	5.2	73.1	17.7	4.0
Mollar	6.3	73.7	19.1	0.9
Mixture	7.4	64.5	19.4	8.7

**Table 6:** Kinetic parameters obtained from the model of independent parallel reactions, as well as the temperature at the maximum rate decomposition (Tm), the weight loss, and the composition of each biopolymer for each variety analysed. HT: high temperatures, LT: low temperatures.

Almond Shell Variety	Component	Ea (kJ·mol^−1^)	K_0_ (s^−1^)	α (kJ∙mol^−1^)	α Ea^−1^ (%)	Tm (°C)	Weight Loss (%)	Amount (%)
Desmayo Rojo	Hemicellulose	132	4.2·10^9^	4.2	3.2	299	25.9	36
	Cellulose	169	1.2·10^12^	0.0	0.0	351	33.8	40
	Lignin LT	170	1.4·10^10^	14.6	8.6	401	6.0	24
	Lignin HT	240	3.2·10^11^	60.0	25.0	445	5.8	
Largueta	Hemicellulose	155	2.9·10^11^	6.6	4.2	308	33.0	44
	Cellulose	202	3.6·10^14^	0.0	0.0	366	28.2	32
	Lignin LT	173	1.6·10^10^	17.9	10.3	398	6.7	24
	Lignin HT	242	8.8·10^10^	63.8	26.4	466	5.7	
Marcona	Hemicellulose	140	1.9·10^10^	4.6	3.3	302	28.9	40
	Cellulose	185	2.3·10^13^	0.0	0.0	356	30.7	36
	Lignin LT	170	1.5·10^10^	13.7	8.1	401	5.8	24
	Lignin HT	242	1.6·10^11^	64.8	26.8	450	6.3	
Mollar	Hemicellulose	140	1.3·10^10^	7.3	5.2	302	28.6	40
	Cellulose	185	2.2·10^13^	0.0	0.0	355	28.6	35
	Lignin LT	171	1.4·10^10^	14.6	8.6	402	6.2	25
	Lignin HT	240	3.2·10^11^	60.0	25.0	445	5.8	
Mixture	Hemicellulose	127	1.2·10^9^	9.1	7.1	287	22.6	37
	Cellulose	173	4.9·10^12^	0.0	0.0	342	19.6	27
	Lignin LT	178	3.1·10^10^	22.0	22.0	389	8.7	36
	Lignin HT	240	3.2·10^11^	65.0	65.0	428	6.7	

**Table 7:** Crystallinity index of different almond shells determined by XRD.

Almond Shell Variety	CrI (%)
Desmayo Rojo	31.3
Largueta	33.8
Marcona	29.7
Mollar	30.2
Mixture	25.1

**Table 8:** Main thermal properties of starch-based polymer/ASP (almond shell powder) biocomposites with different almond shell varieties obtained by differential scanning calorimetry (DSC).

Materials	Melt Enthalphy (J·g^−1^)	Melt Peak Temperature (°C)
As-received Mater-Bi DI01A	28.01	168.84
Biocomposite Mater-Bi DI01A/Desmayo Rojo	21.12	167.91
Biocomposite Mater-Bi DI01A/Largueta	21.31	168.02
Biocomposite Mater-Bi DI01A/Marcona	22.04	168.11
Biocomposite Mater-Bi DI01A/Mollar	21.41	167.95
Biocomposite Mater-Bi DI01A/Mixture	22.03	167.70

**Table 9:** Main thermal properties of starch-based polymer/ASP biocomposites with different almond shell varieties obtained by TGA.

Materials	T_ONSET_ (°C)	T_MAX_ (°C)	Residual Weight (%)
As-received Mater-Bi DI01A	325.98	353.58	2.33
Almond shell	254.67	351.06	22.81
Biocomposite Mater-Bi DI01A/Desmayo Rojo	256.76	304.46	0.63
Biocomposite Mater-Bi DI01A/Largueta	293.91	341.46	0.65
Biocomposite Mater-Bi DI01A/Marcona	281.00	325.27	0.74
Biocomposite Mater-Bi DI01A/Mollar	282.60	318.28	0.83
Biocomposite Mater-Bi DI01A/Mixture	286.19	319.09	0.92
